# Coronary Microcirculation in Aortic Stenosis: A Physiological Hornets’ Nest

**DOI:** 10.1161/CIRCINTERVENTIONS.118.007547

**Published:** 2019-08-16

**Authors:** Hannah Z.R. McConkey, Michael Marber, Amedeo Chiribiri, Philippe Pibarot, Simon R. Redwood, Bernard D. Prendergast

**Affiliations:** 1 Cardiovascular Division, King’s College London British Heart Foundation Centre of Excellence, The Rayne Institute, St. Thomas’ Hospital Campus, London, United Kingdom (H.Z.R.M., M.M., A.C., S.R.R., B.D.P.); 2 Department of Medicine, Institut Universitaire de Cardiologie et de Pneumologie de Québec/Québec Heart and Lung Institute, Laval University, Québec, Canada (P.P.).

## Abstract

Aortic stenosis is a heterogeneous disorder. Variations in the pathological and physiological responses to pressure overload are incompletely understood and generate a range of flow and pressure gradient patterns, which ultimately cause varying microvascular effects. The impact of cardiac-coronary coupling depends on these pressure and flow effects. In this article, we explore important concepts concerning cardiac physiology and the coronary microcirculation in aortic stenosis and their impact on myocardial remodeling, aortic valve flow patterns, and clinical progression.

## Abstract 2

Supplemental Digital Content is available in the text.

“There is a form of cardiac lesion, not infrequent in occurrence, which has a clinical picture so characteristic that it deserves more frequent recognition than it commonly receives.”

Henry A Christian, 18^th^ July 1931^1^

Severe symptomatic aortic stenosis (AS) has a bleak prognosis^2,3^ and no medical treatment exists. As the population ages, the clinical importance and burden of AS are increasing, yet its diagnosis and management are multifaceted, especially in the era of percutaneous interventions. AS is characterized by progressive valve narrowing, which clinically manifests as dyspnea, syncope, and angina despite normal coronary arteries, and patients have a truncated life span of around 2 years without intervention. However, symptomatology is subjective and confounded by comorbidities (particularly in the aging population), and assessment of transvalvular pressures is heavily flow dependent. The clinician is therefore faced with the challenge of evaluating discordant parameters and balancing the potential risks and benefits of valve intervention.

In 1616, William Harvey was the first to propose that blood circulates because of pulsatile cardiac force.^4^ Interactions between the cardiac cycle and coronary circulatory flow were described in 1696 by Scaramucci who suggested that the coronary vasculature is filled in diastole and squeezed empty during systole.^5^ Cardiac-coronary coupling is pertinent in AS because alterations to the coronary microcirculation are synonymous with the pathophysiology of progressive disease. Disruption to the coronary circulation by ventricular hypertrophy, high left ventricular pressure, low coronary perfusion pressure, and extravascular forces (among many other factors) reduce physiological reserve. The ominous symptom of angina correlates with impaired myocardial perfusion reserve and is strongly associated with increased ventricular mass index.^6^ The fact that clinical symptoms occur at the end of the ischemic cascade (whereas perfusion abnormalities can be detected earlier) places great expectation on the physiological evaluation of AS.^7^

Patients with aortic stenosis and an aortic valve area (AVA) < 1 cm^2^ exhibit distinct pathophysiological responses to pressure overload. The ventricle remodels in response to pressure overload in different ways, generating a range of flow and pressure gradient patterns which ultimately cause varying microvascular effects. Detailed understanding of the pressure-flow relationship in this setting is important in fully understanding a patient’s symptoms and the complex relationship between disrupted coronary flow, left ventricular mechanics, and surrogate markers of ischemia.

## Cardiac-Coronary Coupling in Health

**Figure 1.**
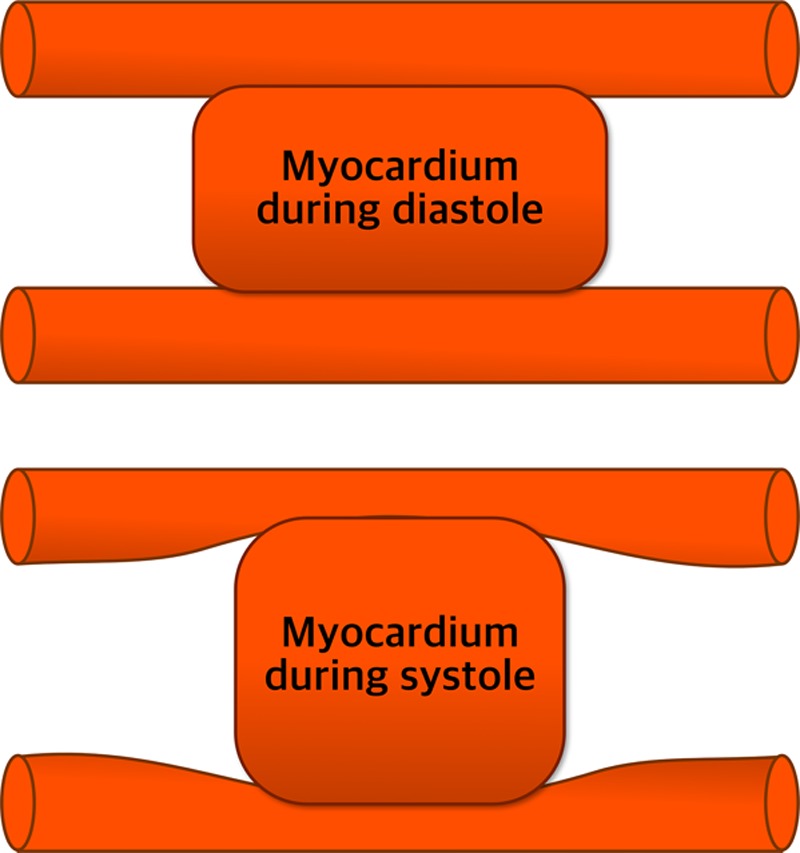
**Myocardial contraction results in muscle shortening and thickening to cause extravascular coronary compression.** The mechanism of myocardium-vessel interaction is a collective effect of contraction-induced intramyocyte pressure and LV pressure-derived interstitial pressure.^12^ Adapted from Westerhof et al^13^ with permission. Copyright ©2006, The American Physiological Society.

Normal resting coronary blood flow comprises around 4% of total cardiac output,^8^ and both oxygen extraction and the myocardial metabolic rate are high when compared with skeletal muscle. During the cardiac cycle, cardiac contraction cyclically increases intramural tissue and microvascular pressures to impede systolic flow. This contraction induces greater subendocardial resistance and blood displacement in comparison with the subepicardium.^9,10^ Once the aortic valve closes and left ventricular (LV) relaxation ensues, the coronary vessels embedded in the myocardium recoil and blood flow accelerates. Coronary flow is dictated by this effect of cardiac contraction—the intramyocardial pump—which pushes blood backward and draws it in during systole and diastole, respectively,^11^ (Figure 1)^12,13^ but is also modulated by aortic and LV pressure, and inotropic state.

**Figure 2.**
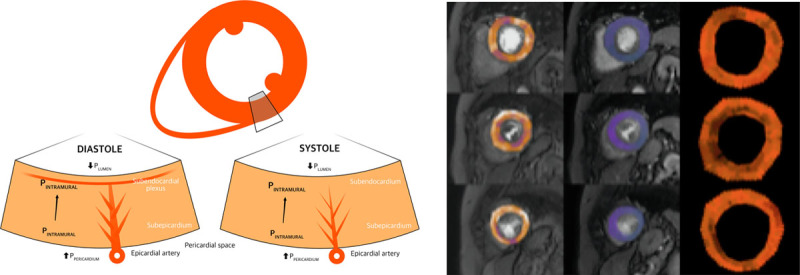
**Structural and functional coronary and myocardial changes during the cardiac cycle and vasodilator stress. A**, Diagrammatic representation of the extravascular forces and intraluminal pressures affecting myocardial layers, demonstrating greater subendocardial contraction during systole. **B**, Perfusion quantification map in a patient with AS (rows from top to bottom are basal, mid, and apical slices, respectively, with stress, rest, and myocardial perfusion reserve [MPR] in columns left to right). Global endocardial-epicardial gradient 0.9, MPR 2.0. P_INTRAMURAL_ indicates intramural pressure; P_LUMEN_, pressure in the left ventricular lumen; and P_PERICARDIUM_, pressure in the pericardial space. Adapted from Duncker and Bache and Bell and Fox^25,26^ with permission. Copyright ©2008, The American Physiological Society.

The waterfall model^14^ proposes that external hydrostatic vascular pressure causes temporary partial collapse of the lumen. Distal luminal pressure therefore becomes similar to external (or intramyocardial) tissue pressure. This external pressure is presumed to result from intraventricular cavity pressure, creating a force against the myocardial walls that reduces from subendocardium to subepicardium. The intramyocardial pump model^15^ expands on this further to allow phase-lag between arterial and venous flows and the role of vascular compliance. Subendocardial vulnerability to ischemia in normal hearts therefore reflects changes in 2 main factors^16^:

1. Increased tension because of systolic compression and increased subendocardial wall stress, accompanied by increased myocardial oxygen requirements.^17^ Both invasive and noninvasive studies have demonstrated increasing intramyocardial pressure from the epicardial to the endocardial surface of the ventricular wall.^18–20^
2. Decreased subendocardial perfusion, secondary to:
  (a) Systolic backflow from endocardial to epicardial vessels causing preferential epicardial blood flow.^21^
  (b) Thinned subendocardial vessel walls relative to their respective subepicardial counterparts^22,23^ making them more prone to external pressure and stress.
  (c) Greater subendocardial vascular volume density^24^—although, with fewer (but larger) perfusion territories, the subendocardium is perfused by a small subset of penetrating arteries (Figure 2).^25,26^

According to Laplace law, circumferential wall tension is equal to the product of the vessel pressure and radius, divided by wall thickness (T=P.r/Th) meaning that the diameter-to-thickness ratio of the vessel or chamber plays an important role. Wall tension and extravascular compressive forces are therefore greatest in the innermost layers of the LV wall. Supporting intramyocardial pressure as a strong determinant of subendocardial blood flow, an early study on anesthetized dogs demonstrated a flow gradient favoring the subendocardium during hyperemia in cardiac arrest (thereby minimizing intramyocardial pressures). However, when tissue pressures were maximized by rapid pacing and coronary perfusion maintained through autoperfusion, the gradient of flow favored the subepicardium.^27^ At low preload, intramyocardial pressure shuts off systolic coronary blood flow across the entire LV wall.^28^ Conversely, there is preferential subepicardial blood flow at high preload.^29^ Coronary blood flow is therefore a balance between intravascular arterial and extravascular tissue pressure.^30^

## Myocardial Blood Supply in Health

The coronary vascular bed acts as the primary gatekeeper to myocardial blood supply. Resting myocardial blood flow (MBF) is the greatest in the subendocardium (endocardial/epicardial flow ratio 1.29–1.35^11,31^), but subepicardial MBF is augmented during adenosine-induced hyperemia to a greater extent. During systole, there is significant subendocardial underperfusion because of the aforementioned physical determinants (transmural perfusion endocardial to epicardial ratio 0.38^11^). After a period of ischemia, reactive hyperemia is earliest in the subepicardium,^9^ and this delayed subendocardial response is thought to be because of sluggish reopening of the coronary vasculature embedded in ischemic, poorly compliant myocardium.

Among many other mechanisms, the gradient in coronary perfusion pressure (difference between aortic and LV end diastolic pressure) facilitates coronary perfusion, and flow is determined by the product of the net velocity-time integral and cross-sectional arterial area (Q=VA). The largest cross-sectional area exists in the microvasculature where reduced velocity allows adequate time for capillary bed gas transfer. In normal hearts, aortic and LV pressures are coupled during systolic ejection and higher perfusion pressure gradients enable coronary perfusion during diastole. There is a nonlinear connection between cross-sectional area and transmural pressure because vascular tone is influenced by metabolic/neurohormonal mediators and physical forces. According to Ohm’s law, flow through a vascular bed is equal to the perfusion pressure gradient divided by vessel resistance, 8ηl/πr^4^ (Hagen-Poiseuille equation, where η is blood viscosity, l is vessel length, and r is vessel radius). Microvascular resistance is therefore primarily determined by lumen diameter and vasodilatation is the principle means of microcirculatory autoregulation.

During maximal coronary vasodilatation, coronary flow depends on the relative duration of diastole.^32^ This diastolic time fraction (the length of diastole/length of cardiac cycle) has an inverse relationship with heart rate and is also determined by other modulators of systolic duration (such as altered myocyte contraction). Decreased coronary perfusion pressure induces an increase in diastolic time fraction, which in turn reduces the duration of intramyocardial vessel compression.

## Coronary Wave Intensity Analysis

**Figure 3.**
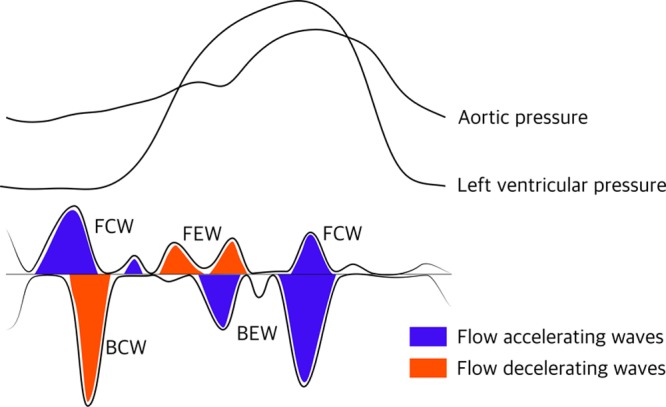
**The 4 dominant coronary waves during the cardiac cycle in relation to hemodynamic indices (not to scale).** BCW indicates backward compression wave; BEW, backward expansion wave; FCW, forward compression wave; and FEW, forward expansion wave.

Studies of wave intensity analysis have identified 4 main coronary waves within the cardiac cycle in health and disease^33^ (Figure 3).

Quantification of net wave intensity through the product of changes in pressure and flow velocity makes it possible to segregate components of coronary flow into forward or backward traveling waves from the aorta or microcirculation, and those caused by suction (expansion) or compression—blood can be pushed into or pulled out of the coronary circulation. Flow from the coronary circulation to the myocardium is largely determined by the prominent backward expansion wave (BEW), originating at the onset of LV relaxation. The decelerating backward compression wave and forward expansion wave impede coronary flow, while the BEW and forward compression wave are accelerating waves. Information concerning the size, direction, and duration of coronary waves throughout the cardiac cycle has helped us understand coronary flow in normal hearts, in AS,^19^ and transcatheter aortic valve implantation (TAVI),^34,35^ hypertrophic cardiomyopathy^36^ and several other settings.^33,37–42^

## Cardiac-Coronary Coupling in AS

The pathophysiology of calcific degenerative AS has 2 distinct phases: initiation and propagation.^43^ The former overlaps with the development of atherosclerosis, centered around endothelial disruption and activation of inflammatory responses. Progressive AS induces left ventricular hypertrophy (LVH) to increase contractile force and reduce wall stress^44^ in response to progressive and eventually insurmountable afterload. Compressive forces resulting from rising intracavitary pressure determine coronary perfusion pressure and limit coronary circulatory response to increased myocardial demand—an association related to the extent of LVH.^45^ Oxygen requirements increase while perfusion through the small perforating coronary network is compromised by fixed elevated systolic wall stress^46,47^ and reduced relative capillary density,^48,49^ creating supply-demand mismatch. These structural changes of vascular rarefaction, compressive forces, and perivascular fibrosis and functional changes, such as reduced diastolic perfusion time (DPT, defined as [RR interval]−[S_1_-S_2_ interval]×heart rate) and endothelial and smooth muscle dysfunction, all exert adverse effects.

Preferential coronary flow shifts from the endocardium to epicardium resulting in a significant decrease in subendocardial (but not subepicardial) MBF.^50^ This reversal of normal endocardial-epicardial blood flow ratio^51^ at rest is fundamental to the pathophysiology of AS, resulting in subendocardial ischemia,^52^ apoptosis,^47^ and fibrosis—clinically manifest as angina despite normal epicardial coronary arteries. Noninvasive detection of this shift in resting endocardial-epicardial ratio could be used to guide timing of valve intervention.

Severe AS exhibits an array of flow parameters, but there is significant LV outflow tract obstruction in all forms, typically accompanied by LVH,^53^ which may cause dynamic obstruction in late systole with systolic anterior motion of the mitral valve. Unlike hypertrophic cardiomyopathy, where there is a strong linear relationship between peak-to-peak gradient and peak instantaneous gradients, significant scatter exists in AS patients.^54^

One study demonstrated that severity of AS and parameters of LV workload (but not LVH or diastolic indices) have important roles in determining coronary flow reserve (CFR).^55^ Another study, however, correlated impaired perfusion reserve with valve stenosis, myocardial fibrosis, and strongly with LVH.^45^ Cardiac amyloid is common in this population and may confound results.

There are strong similarities in the pathogenic manifestations of AS and hypertension, that is, interstitial and perivascular fibrosis, cardiomyocyte hypertrophy, reduced DPT, increased diastolic filling pressure (compressing the endocardium) and diastolic dysfunction, capillary rarefaction,^51^ and arteriolar remodeling.^56^ However, key differences exist. The BEW is the most important contributor to coronary blood flow and a measure of microcirculatory function—it is increased at rest in AS^34,35^ but reduced in isolated LVH,^33^ probably as a result of lower wall stress and slower isovolumetric LV relaxation (dP/dt_min_). Furthermore, there is a direct relationship between systolic coronary velocity and systolic perfusion pressure in hypertensive patients with no AS—extravascular compressive forces which normally impede systolic coronary flow may be overcome in the setting of higher perfusion pressure.^57^

After TAVI or surgical aortic valve replacement, there is restoration of myocardial perfusion, oxygenation, energetics, and contractility, accompanied by improved microcirculatory function as a result of the relief of mechanical obstruction and wall stress, and eventual LVH regression.^58,59^ Indexed stroke volume drops sharply (41±8 to 33±10 mL/m^2^; *P*<0.001) as a result of increased systemic vascular resistance (*P*<0.0001), despite no clear difference in global afterload measured by valvulo-arterial impedance (Zva).^60^ Hyperemic microvascular resistance (hMR) decreases after TAVI, independent of resting hemodynamics.^61^ Remaining hypertrophy continues to influence coronary physiology with improved (but not normalized) CFR.

## Disrupted Coronary Flow in AS

**Figure 4.**
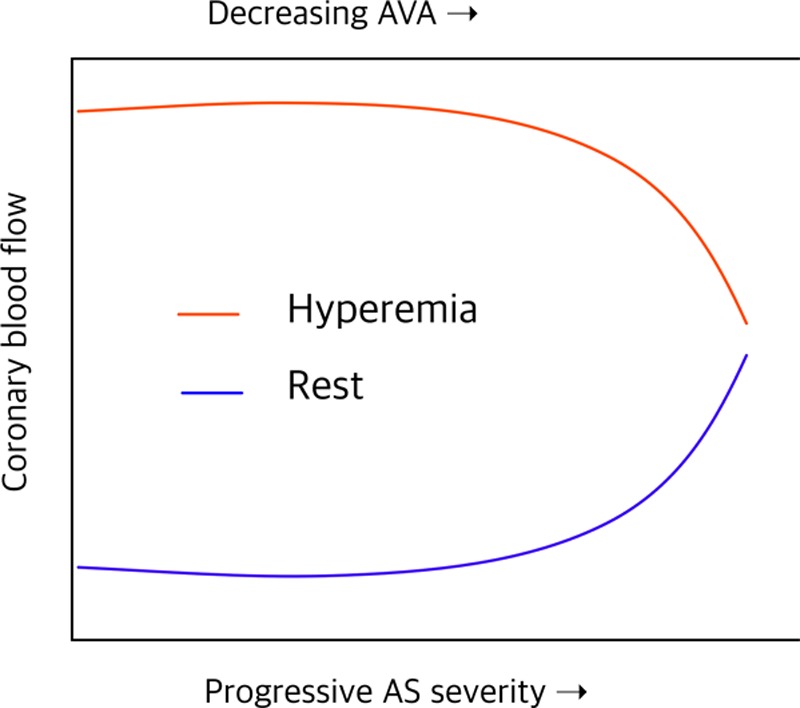
**Impairment of coronary flow reserve in progressive aortic stenosis (AS): simulated resting and hyperemic mean coronary blood flow as a function of the severity of AS and estimated orifice area.** Induced hyperemia is fundamentally important during circulatory assessment in AS because adaptive hyperemia is already established at baseline—several well-cited studies are flawed in this respect. AVA indicates aortic valve area. Adapted from Garcia et al^65^ with permission. Copyright ©2009, The American Physiological Society.

Microcirculatory autoregulation induces vasodilation to minimize microvascular resistance and increase total resting MBF, resulting in reduced CFR^62,63^ and MPR^64^ because of paired inability to further vasodilate (Figure 4^65^). Low coronary perfusion pressure,^66^ extravascular compressive forces,^67^ and reduced DPT^46,56,61^ all seem to play a role. Reduced DPT because of prolonged systole in AS supports the maldistribution theory.^68^

In contrast to normal physiology, the relative contribution of accelerating waves to total wave intensity decreases with exercise and hyperemia in AS.^19^ The contrary is true for decelerating waves: the backward compression wave increases with exercise and hyperemia, thereby hampering flow and driving ischemia. Davies et al^34^ analyzed wave intensity in the left main stem at programmed heart rates before and after TAVI (albeit without inducing hyperemia) and demonstrated progressive reduction (rather than the expected increase) in the BEW with increasing heart rate. This paradoxically blunted microvascular response normalized after TAVI where induced tachycardia caused the BEW to increase rather than decrease, probably because of a sharp reduction in afterload. A chronological summary of relevant coronary physiology and aortic stenosis studies is displayed in Table I in the Data Supplement.

Before valve intervention, forward flow is delayed, and peak systolic flow and velocity-time integral reduced.^69^ In comparison to normal hearts, the aortic-ventricular diastolic relationship impairs coronary perfusion.^34,70^ After TAVI, however, all coronary waves augment (apart from the backward compression wave^35^), inducing an immediate increase in coronary flow.^71^ In particular, the forward compression wave improves and its onset is shortened.^35^ Increased aortic diastolic pressure (with consequent forward pressure at the coronary ostia) accompanied by decreased LV end diastolic pressure and increased DPT causes an elevated driving pressure across the coronary bed. In part, improved forward flow may be because of the resolution of abnormal helical and eccentric vertical flow patterns seen in AS,^72^ which reduce high fluid pressure and the associated Venturi effect in the proximal aorta and coronary ostia.

LV systolic wall stress index and peak systolic flow velocity^73^ are tightly knit, suggesting that extravascular compressive forces change systolic flow, although these changes are independent of LV mass. This may explain why CFR may not respond immediately to relief of valve obstruction but improves after 1 year.^74^ Other studies have also demonstrated improved subendocardial blood flow at 2 weeks,^50^ CFR at 6 months,^75^ and indexed myocardial perfusion reserve at 8 months^45^ after valve replacement. The evidence is strong for structural and hemodynamic effects as the cause of myocardial ischemia in AS.

The pathophysiological and clinical manifestations of coronary microvascular dysfunction, described as heightened sensitivity to vasoconstrictor stimuli associated with limited vasodilator capacity, have been previously classified^56^ (Table).^66^

**Table.**
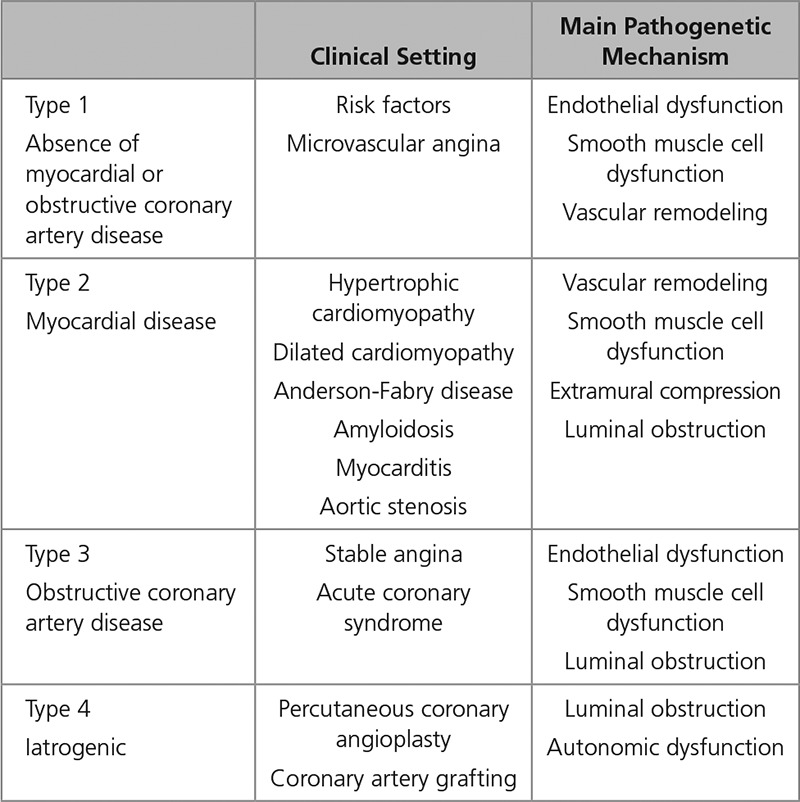
Classification of Coronary Microvascular Dysfunction^66^

Coronary physiological response to hyperemia can also be grouped into 4 categories, depending on the presence of normal or abnormal CFR (>2.0 and <2.0, respectively) and normal or abnormal hMR (<1.7 and >1.7 mmHg/cm per second, respectively).^76^ The reference standard of microvascular dysfunction is invasive measurement of coronary vascular resistance using pressure and flow during hyperemia,^77^ where hMR is calculated by dividing the mean distal coronary pressure (Pd) by the hyperemic average peak Doppler flow velocity. However, hMR does not determine global microvascular dysfunction but minimal static resistance which is strongly dictated by microcirculatory remodeling—either intrinsic (arteriolar remodeling or capillary rarefaction) or extrinsic to the vascular tree.

Two reasons for reduced CFR in AS have been proposed. The first hypothesis is that inherent microvascular dysfunction elaborates ischemia, as initially proposed by Ahn et al^6^ who demonstrated reduced myocardial perfusion reserve in patients with AS and angina using perfusion cardiac magnetic resonance imaging (without reporting hemodynamic or microvascular mechanisms).^77^ The second is that ischemic signs and symptoms result from high wall stress and mechanical effects in response to AS, supported by improvement of coronary physiological indices immediately after TAVI.

**Figure 5.**
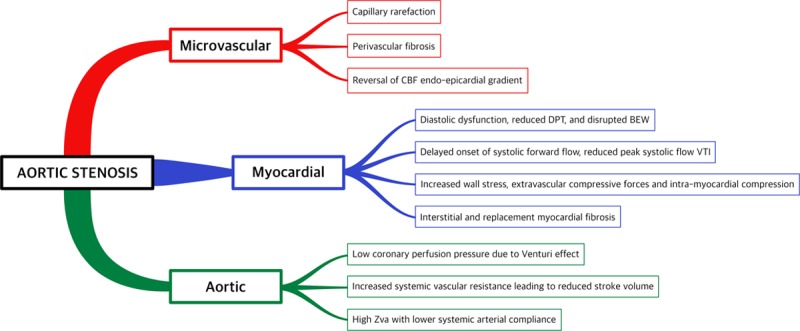
**Factors implicated in disrupted coronary flow and reduced coronary flow reserve in aortic stenosis.** Compensatory mechanisms fail because of structural and mechanical effects on the ventricle and coronary circulation. There is reduced physiological reserve as a result of inadequate myocardial oxygen supply and increased oxygen demand. BEW indicates backward expansion wave; CBF, coronary blood flow; DPT, diastolic perfusion time; and VTI, velocity-time integral.

Transmural CFR and subendocardial-to-subepicardial perfusion ratio fall directly with decreased hyperemic DPT in AS (measured using positron emission tomography) and improve with increased hyperemic DPT and increased AVA after surgical aortic valve replacement,^46,74^ supporting a prominent role for hemodynamic conditions in determining CFR—microvascular disease would be expected to yield uniformly reduced transmural perfusion without a gradient.^77^ Equally, myocardial perfusion reserve may be low in AS patients^6^ because of the resting increase in perfusion (rather than reduced stress perfusion) because since myocardial perfusion reserve is a relative ratio of stress-to-rest of the magnetic resonance signal,^77^ and independently associated with exercise capacity.^64^ Intrinsic endothelial dysfunction does not correlate convincingly with hemodynamic factors that are promptly corrected after TAVI^61^—proposed mechanisms impacting disrupted microvascular function are illustrated in Figure 5.

Lumley et al^19^ found that perfusion efficiency during exercise in patients with AS was reduced when compared with normal patients, as a result of augmented early systolic deceleration waves (backward compression wave) and attenuated rise in systolic acceleration waves (forward compression wave). Importantly, further assessment found that AS patients and those with normal hearts are able to reduce microvascular resistance to the same extent.^19^ Decreased hMR after TAVI independent of resting hemodynamics has also been demonstrated in patients with severe AS (not differentiated into flow or pressure gradient status).^61^ Clearly, both intra- and extra-myocardial pressures dictate coronary supply and a combination of factors is likely to be responsible for the distortion of coronary flow and impaired CFR in AS.

## Aortic Valve Flow and Pressure Gradients

**Figure 6.**
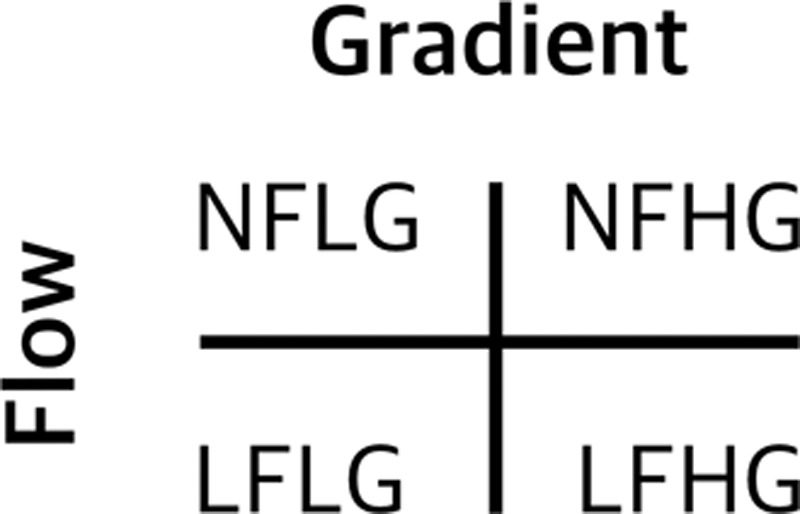
**Classification of aortic stenosis according to flow (low-flow <35 mL/m^2^, normal-flow >35 mL/m^2^) and gradient (low-gradient mean pressure gradient [MPG] <40 mmHg, high-gradient MPG >40 mmHg).** Low-flow low-gradient can be further subdivided into classical and paradoxical according to the presence or absence of impaired left ventricular function. LFHG indicates low flow-high gradient; LFLG, low flow-low gradient; NFHG, normal flow-high gradient; and NFLG, normal flow-low gradient.

**Figure 7.**
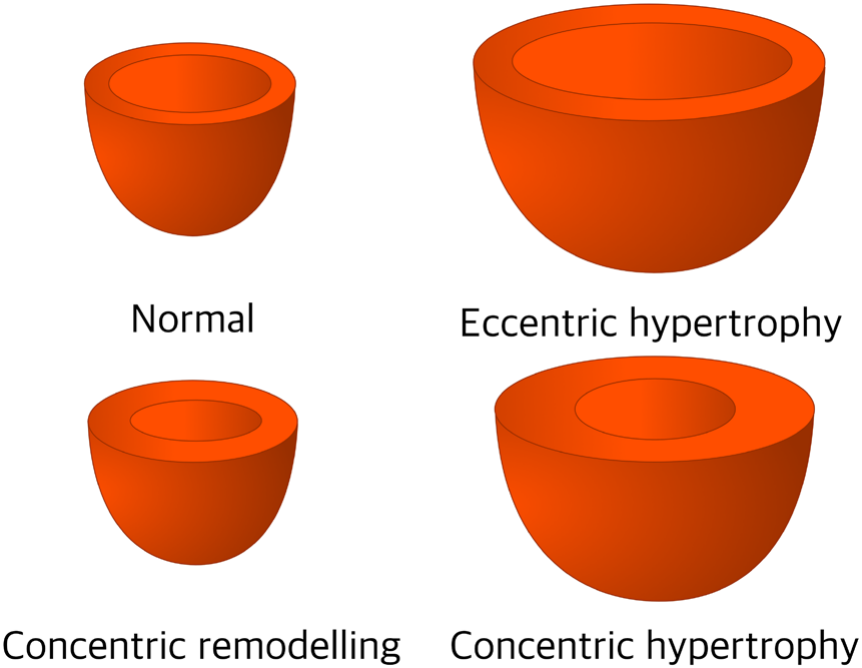
**Patterns of cardiac remodeling based on normal or increased mass to volume ratio (concentric remodeling and concentric hypertrophy) and normal left ventricular wall thickness (concentric remodeling) or hypertrophy (concentric and eccentric).** Adapted from Gjesdal et al^78^ with permission. Copyright ©2011, Springer Nature.

The adaptive compensatory response to AS ultimately become maladaptive and results in cardiac decompensation, yet there are several guises with distinct anatomic and physiological characteristics (Figures 6 and 7).^78^ Normal-flow high-gradient AS usually provokes concentric hypertrophy, whereas paradoxical low-flow low-gradient (pLFLG) AS patients demonstrate concentric remodeling.^79^

The ventricular adaptive response to high afterload in combination with valve obstruction is poorly understood and may be more varied than is currently appreciated. Flow and stroke volume can both be reduced or normal in patients with preserved and reduced LV ejection fraction (LVEF).^80^ While there is clear consensus that symptomatic AS with AVA <1cm^2^, peak velocity (V_max_) >4 m/s, and mean pressure gradient >40mmHg warrants intervention, diagnostic ambiguity exists in patients with a small AVA and lower pressure gradients (despite preserved LVEF) where lower stroke volumes contribute significantly to discrepancies.^81^ Aging, hypertension, diabetes mellitus, and dyslipidemia are associated with microvascular dysfunction and impaired CFR, and there is a higher proportion of diabetes mellitus and hypertension in pLFLG cohorts. These, in turn, are associated with an intrinsic likelihood of impaired CFR,^82–84^ arising as a consequence of nonendothelium-dependent disorders of nitric oxide metabolism, dysregulation of inflammatory cytokines, estrogen, or adrenergic receptors, and alterations in expression or production of local vasoactive substances such as angiotensin II and endothelin.^66^

Low-gradient groups may be more susceptible to microvascular disturbance, as evidenced by a higher burden of subendocardial fibrosis on cardiac magnetic resonance.^85^ Since the first description of pLFLG AS by Hachicha et al,^86^ there have been conflicting reports and evidence concerning the underlying pathophysiology. Accounting for up to 35% of severe AS cases (with a female preponderance), many are undiagnosed and surgical referral is frequently delayed or overlooked. The syndrome entails the perfect storm of valve, ventricular, and vascular abnormalities, with valve stenosis, concentric LV remodeling (culminating in restrictive physiology), and high Zva with markedly lower systemic arterial compliance and higher arterial resistance.^85–90^

A low-indexed stroke volume predicts mortality and risk increases sharply when it is <35 mL/m^2^.^91–93^ Although still controversial, the bulk of evidence suggests that patients with AS and SVi <35 mL/m^2^ have markedly worse outcomes.^82,86–88,92,94–106^ Some discrepant studies (which include a high proportion of asymptomatic patients or fail to account for stroke volume)^107–110^ have been criticized for imprecise data analysis and misclassification.^111^ The phenomenon of distinct remodeling is poorly understood, and there is a paucity of invasive data to characterize the cohort and understand factors that predict poor outcome and the response to valve intervention.

European^112^ and American^113^ guidelines provide a Class IIA indication for aortic valve intervention in symptomatic pLFLG AS but only after careful confirmation of clinical, hemodynamic, and anatomic data (in the normotensive setting), and exclusion of pseudo-stenosis, where the myopathic ventricle fails to generate adequate force. Although survival is improved when it is treated,^80,99,105,106,114,115^ these patients have adverse outcomes during and after valve intervention when compared with other AS cohorts,^82,100,106^ perhaps related to the burden of myocardial fibrosis.^116,117^ This fibrosis also impacts on myocardial perfusion reserve owing to reduced arteriolar and capillary density.

## Structural Remodeling in Low-Gradient AS

The complex collagen weave is responsible for much of the ventricle’s passive diastolic stiffness,^118^ and remodeling in response to pressure overload causes fibroblast proliferation and collagen I accumulation.^119^ Myocardial collagen deposition is a common end point of many pathologies and accompanies advanced aging.^120^ Myocardial hypertrophy is detrimental to overall survival^121–123^ and correlates with fibrosis, impaired longitudinal shortening, and worsening diastolic function. This fibrosis associated with AS^124–127^ is a crucial determinant of cardiac dysfunction and prognosis,^116,124,125,128,129^ and replacement fibrosis may be the result of myocyte apoptosis accounting for progression to heart failure.^130^ Interstitial, subendocardial, and mid-wall patterns of fibrosis have been demonstrated in patients with AS and normal coronary arteries.^85,116,117,123,131–137^

While endomyocardial biopsy is the gold standard for confirming fibrosis,^138^ cardiac magnetic resonance imaging has been widely used in its detection, either using T1 mapping to calculate extracellular volume fraction or late gadolinium enhancement. Extracellular volume fraction can detect extracellular volume expansion with diffuse fibrosis, whereas late gadolinium enhancement only identifies replacement fibrosis.^139^

Patients with pLFLG AS typically have more profound impairment of LV longitudinal function^98,114,140–142^ and more florid myocardial fibrosis, predominantly located in the subendocardium.^85^ In comparison to circumferential fibers located in the mid-wall, longitudinal subendocardial fibers (responsible for long-axis function)^143–146^ are particularly vulnerable to microvascular ischemia and wall stress.^85,131^ Impaired longitudinal function as a consequence of subendocardial injury, small LV cavity size, and increased wall thickness lead to reduced stroke volume and lower flow-dependent valve gradients.^147^ Reduced stroke volume is primarily because of deficient LV filling (rather than emptying),^95^ and preserved LVEF should not be construed as normal systolic function. Consistent with this theme, a recent study demonstrated that indexed AVA, female sex, an abnormal exercise ECG and myocardial perfusion reserve (but not valve gradients or LV function) were independent predictors of event rates in moderate-severe AS.^148^

This distinct remodeling may be explained by decreased cardiac reserve resulting from chronic exposure to high afterload, eventually exceeding the limit of compensatory mechanisms with resulting LV impairment and reduced cardiac output.^86^ It is also possible that these patients have a coexisting or secondary heart failure syndrome, akin to heart failure with preserved ejection fraction,^149^ the cause of which is complex and poorly understood. Importantly, these 2 pathologies (which are both relatively common in older age) are not mutually exclusive and exhibit significant similarities, including impaired LV relaxation and microvascular abnormalities.^46,73,150–153^ Indeed, galactin-3, a novel marker of myocardial fibrosis, has prognostic value in heart failure with reduced or preserved ejection fraction^154,155^ and is associated with adverse outcomes after TAVI^156^—despite the lack of any association with AS severity.^157^ Patients with elevated galactin-3 before TAVI have lower valve gradients and reduced LVEF (although data were not divided into AS cohorts).^156^ Similarly, 1 study revealed that low flow (but not low LVEF or low gradient) is an independent predictor of early and late mortality after TAVI in high-risk AS patients.^100^ Comparable to patients with heart failure, LVEF does not correlate with outcomes.

Equally, the peril of low flow does not correlate with aortic valve calcification. There is less aortic valve calcification but higher global afterload in pLFLG than other types of AS,^80^ suggesting a coexistent ventricular disease entity that may explain why these patients have reduced survival benefit after valve intervention than other subgroups. This would support the theory that pLFLG AS is not end-stage normal-flow high-gradient AS^158^ but a distinct and separate entity.^159–161^ Furthermore, the concept of pLFLG AS as a transition stage from nonsevere to severe^80^ is undermined by a preponderance of myocardial injury and adverse outcomes.

## Clinical Implications of Impaired Coronary Flow

Reduced capacity to augment myocardial oxygenation in response to stress is a physiological hallmark of AS and manifest by angina, dyspnea, and syncope. Up to 40% of patients with AS experience angina despite normal coronary arteries^162^ and are at increased risk of sudden death.^163^ These patients have reduced MBF, impaired CFR, and increased apoptosis^47^ and are more likely to have impaired reserve^6,162^ and diminished exercise capacity.^64^ One study found that low CFR was the only independent predictor of future cardiovascular events in AS patients.^164^ Exertion accentuates the imbalance between supply and demand, and rising LV end diastolic pressure blunts the pressure gradient required to achieve adequate coronary perfusion. Any rise in LV end diastolic pressure or fall in AVA has a deleterious effect on coronary supply,^35,46^ and there is a strong association between ventricular load (measured by LV rate-pressure product) and decreased CFR, particularly affecting the subendocardium.^46^ Stuttering ischemia yields subclinical LV dysfunction and apoptosis, which is linked with myocardial fibrosis^165^—an independent predictor of mortality.^116^

Biomarkers have an emerging role in the assessment of asymptomatic AS.^166^ High-sensitivity troponin I correlates with LVH, fibrosis, and clinical event rates,^134^ while cardiac myosin-binding protein C correlates closely with LV mass, fibrosis, and all-cause mortality (but not valve gradient).^167^ BNP (NT-pro B-natriuretic peptide) levels are significantly higher in paradoxical and classical low-flow low-gradient AS,^85^ and correlate with CFR ≤2.5 and parameters of diastolic function^168^—use of BNP in asymptomatic AS is endorsed by recent European guidelines.^112^

## Conclusions

Patients with AS host a caustic environment where impaired microvascular responses are compounded by high wall stress and hemodynamic load; those with angina (and impaired CFR) are at increased risk of sudden death. Progression of AS is characterized by discrepancies between blood supply and metabolic demand. There is an array of abnormalities in myocardial remodeling, stroke volume, pressure gradients, and disordered coronary flow, which contribute to the signatures that determine varying AS phenotypes. These distinctions, which correlate with clinical outcomes, should prompt a directive path of physiological research. All patients with AS are not equal and the optimal timing and modality of treatment might differ according to phenotype. Relying on peak velocity to determine severity is now obsolete. Timing of intervention is crucial in avoiding irreversible myocardial fibrosis and a burnt out ventricle. Assessment of microcirculatory function may hold the key.

## Sources of Funding

H. McConkey is supported by a Clinical Research Training Fellowship grant from the British Heart Foundation (FS/16/51/32365).

## Disclosures

None.

## Supplementary Material
