# Supplementary material for: Coronary Microcirculation in Aortic Stenosis: A Physiological Hornets’ Nest
Source: Circ Cardiovasc Interv. 2019 Aug 16;12(8):e007547. doi: 10.1161/CIRCINTERVENTIONS.118.007547 (PMC6733603; doi:10.1161/CIRCINTERVENTIONS.118.007547)
Supplement: Supplementary file 1 [file hcv-12-e007547-s001.pdf]

SUPPLEMENTARY MATERIAL

Supplementary Table 1: Reported comparisons of physiological measures in AS (presented in chronological order)

	CORONARY INDICES				SYSTEMIC/VALVE HEMODYNAMIC INDICES		MYOCARDIAL INDICES		ISCHEMIC/OTHER INDICES
**Fallen EL et al 1967[1]**									
**Left heart and coronary sinus catheterization with baseline and hyperemic measurements using Isoproterenol in 18 patients with severe AS (7 patients without angina, 5 patients with angina but without CAD, and 6 patients with angina and CAD)**									
	**Hyperemic CBF**						**Hyperemic myocardial oxygen extraction**		**Lactate production during hyperemia**
No angina	+						-		o
Angina without CAD	o						o		+
Angina with CAD	+						-		+
**Marcus ML et al 1982[2]**									
**Coronary reactive hyperemia response following 20 second LAD occlusion in 14 symptomatic severe AS patients during SAVR**									
	**Peak-resting velocity ratio**		**Repayment- debt area ratio**						
Controls	o		o						
Severe AS	-		-						
**Omran H et al 1996[3]**									
**TEE Doppler of the LAD in 46 patients (34 symptomatic, 12 asymptomatic) with moderate-severe AS**									
	**Peak coronary systolic velocity**	**Peak coronary diastolic velocity**	**Systolic VTI**	**Diastolic acceleration time**	**AVA**	**Pressure gradient**	**LVMI**	**LV wall stress**	
Controls	o	o	o	o			o		
All AS	-	-	-	+			+		
Symptomatic vs asymptomatic AS	Lower	Higher	Smaller	Longer	Smaller	Higher	Higher	Higher	
**Julius BK et al 1997[4]**									
**Invasive rest and dipyridamole stress data from patients with severe AS and angina (n=18), without angina (n=11) and control patients (n=7)**									

	**CFR**	**Coronary resistance per 100g LVMM**		**LV peak systolic pressure**		**Mid-wall stress**	**Resting CS blood flow**	**Max CS blood flow**	**ST depression on exercise electrocardiogram**	
Controls	o	o		o		Low	o	o		
AS - angina	-	o		+		Moderate	+	-		
AS + angina	--	o		++		High	+	--	More pronounced	
**Hildick-Smith et al 2000[5]**										
**Echocardiographic rest and hyperemic LAD Doppler data from 24 patients with severe AS pre- and 6-months post-SAVR**										
	**CFR**	**Hyperemic peak systolic velocity**	**Hyperemic peak diastolic velocity**			**LVMI**				
Pre-SAVR	-	-	-			+				
Post-SAVR	o	o	o			o				
**Rajappan et al 2002[6]**										
**CMR, ECHO and PET data from 20 patients with moderate-severe AS (asymptomatic and symptomatic)**										
	**CFR**			**AVA**	**hDPT**	**hTransmural MBF**				
Controls	o					o				
Mod-severe AS	-			Increase linearly related to hMBF	Significant correlation with hMBF and CFR	-				
**Davies et al 2006[7]**										
**Invasive coronary physiology at the time of angiography in 20 patients without aortic stenosis (10 controls, 10 with LVH)**										
	**Mean CBFV**	**BEW**	**BEW/FCW**							
Controls	o	o	o							
LVH	o	-	-							
**Galiuto et al 2006[8]**										
**Contrast and Doppler echocardiographic in 11 patients with severe symptomatic AS awaiting SAVR, LV biopsy during SAVR**										
	**Baseline CBFV**	**CBFVI**	**CFR**			LVMI			**SI*β**	**Apoptosis**
Controls	o	o	o			o			o	o
Severe AS + LVH	+	-	-			+			-	+
**Davies et al 2011[9]**										

Invasive coronary physiology at baseline and during rapid pacing before and after TAVI in 11 patients with severe symptomatic AS

	BEW	Flow velocity	Pressure time integral
Pre-TAVI baseline	+	o	o
Pre-TAVI 120bpm	o	o	-
Post-TAVI baseline	o	o	o
Post-TAVI 120bpm	+	o	-

Steadman et al 2012[10]

Cardiopulmonary exercise testing, CMR and echocardiography in 46 patients with severe AS awaiting SAVR (9 asymptomatic, 37 symptomatic)

	Peak AVG	MPR	LVMI	Septal E/e'	Resting MBF	LGE
Association with perfusion reserve (p value)	0.02		<0.001	0.03		0.002
Association with peak VO_2 (p value)	0.24	0.004	0.89	0.02	0.001	0.73

Mahmod M et al 2014[11]

CMR in 28 patients with severe AS (3 asymptomatic, 25 symptomatic) - 14 of the 25 symptomatic patients were rescanned 8 months after SAVR

	MPRI	Circumferential strain	Blood O2 level dependent signal intensity change	PCr/ATP
Controls	o	o	o	o
Severe AS	-	-	-	-
Post-SAVR	o	o	o	o

Ben-Dor I et al 2014[12]

Doppler LAD flow using TEE during TAVI in 90 patients with severe AS

	Peak systolic coronary velocity	Peak diastolic coronary velocity	Systolic VTI	Diastolic VTI
Pre-TAVI	o	o	o	o
Post-TAVI	+	+	+	+

Wiegerinck et al 2015[13]

Invasive coronary physiology immediately pre- and post-TAVI in 27 patients with severe AS)

	bCFV	hCFV	CFR	bMR	hMR	hAPV

Controls	o	o	o	o	o	o							
Pre-TAVI	+	-	-	-	o	-							
Post-TAVI	+	o	o	-	-	o							
**Gutierrez-Barrios A et al 2015[14].** **Invasive coronary physiology in 35 patients with severe AS (unknown symptoms)**													
	**IMR**						**AVA**		**LVMI**			**BNP**	
Correlation with CFR (1.39+/-0.57)	r=-04, p0.03						CFR < 2 0.63+/-0.05 p0.04		r=0.38, p0.08			r=-0.38, p0.02	
**Ahn JH et al 2016 [15]** **CMR in 84 patients with severe AS (43 with angina, 41 without angina)**													
									**MPRI**	**LVMI**	**CI**	**LGE**	
Controls									o	o	o	o	
AS - angina									-	+	+	+	
AS + angina									--	++	++	+	
**Rolandi et al 2016[16]** **Invasive coronary physiology immediately pre- and post-TAVI in 15 patients with severe AS**													
	**CFR**	**bBEW**	**hBEW**	**Coronary pulse pressure (bPPd and hPPd)**	**Systolic VTI rest and hyperemia**								
Controls	o	o	o	o	o								
Pre-TAVI	-	+	-	o	+								
Post-TAVI	-	++	o	+	++								
**Singh et al[17]** **Exercise test, echocardiography and CMR in 174 asymptomatic patients with moderate-severe AS**													
							**AVA**	**AVG**	**Global MPR**	**Stroke volume**	**LVMI**	**Fibrosis**	**NT-proBNP**
No event							o	o	o	o	o	o	o
Event							-	+	-	-	Predicted outcome	o	+
**Lumley et al 2016[18]**													

**Rest and exercise coronary physiology in 22 patients with severe symptomatic AS and 38 healthy controls with stress echocardiography in 13 AS patients**									
	**Hyperemic CBF**	**Fall in MR with hyperemia**	**Exercise CFR**	**Hyperemic CFR**			**Myocardial workload at rest**	**Myocardial workload during exercise**	
Controls	o	-	o	o			o	o	
AS patients	-	--	o	-			+	+	
**Gutiérrez-Barrios et al 2017**[19] **Rest and hyperemic coronary physiology in 36 patients with severe AS and 10 healthy controls**									
	**CFR**	**Tmn_{rest}**	**Tmn_{hyp}**	**IMR**	**Br**				
Controls	o	o	o	o	o				
AS patients	-	Faster	Slower	+	-				

<u>Online Table 1</u>: Comparison of outcomes from relevant studies.

Abbreviations: AS: aortic stenosis, AVA: aortic valve area, AVG: aortic valve gradient, BEW: backward expansion wave, bMR: baseline microvascular resistance, BNP: b-type natriuretic peptide, bPPD: baseline coronary pulse pressure in diastole, Br: baseline resistance, CBF: coronary blood flow, CBFV: coronary blood flow velocity, CBFVI: coronary blood flow velocity indexed for LV mass, CFR: coronary flow reserve, CI: cardiac index, CMR: cardiac magnetic resonance, CS: coronary sinus, ECHO: echocardiography, FCW: forward compression wave, hAPV: hyperemic average peak flow velocity, hDPT: hyperemic diastolic perfusion time, hMR: hyperemic microvascular resistance, hPPd: hyperemic pulse pressure in diastole, hTransmural MBF: hyperemic transmural myocardial blood flow, IMR: index of microvascular resistance, LAD: left anterior descending artery, LGE: late-gadolinium enhancement, LVMI: indexed left ventricular mass, LVMM: left ventricular muscle mass, MPR: myocardial perfusion reserve, MPRI: indexed myocardial perfusion reserve, PCr/ATP: phosphocreatine/adenosine triphosphate, PET: positron emission tomography, PW: pulse wave spectral Doppler, SAVR: surgical aortic valve replacement, SI*β: peak signal intensity (SI)

multiplied by the rate of signal rise, TEE: transesophageal echocardiography, Tmn_{hyp}: transit mean times during hyperemia, Tmn_{rest}: transit mean times at rest, Zva: valvulo-arterial impedance.

Physiological assessment of coronary stenoses in the setting of AS

Symptomatology overlaps in patients with AS and epicardial coronary disease, and distinction may be clinically impossible. Physiological assessment of epicardial coronary stenoses in this setting is challenging due to compounding factors that contribute to myocardial ischemia, such as LVH and excess afterload. Functional evaluation of isolated coronary artery disease is well validated[20,21] and strongly linked with clinical outcomes but a clear understanding of the pitfalls amidst AS is important for clinicians.

The results of coronary physiological assessment should be interpreted with caution in AS since it is not a true resting state. Distorted values may be caused by several factors:

1. Elevation of coronary sinus outflow and distal coronary pressure[22] which may underestimate the significance of a coronary stenosis
2. Secondary LVH which causes reduced capillary density and abnormal vasoreactivity[23]
3. Elevated right atrial pressure[24]

LVH causes fixed elevation of coronary resistance which may also be increased by neurohumoral factors that influence the response to adenosine[25] - these include α-adrenoceptor agonists, angiotensin and vasopressin[26], the levels of which may be modulated by medication - adenosine infusion is safe and well-tolerated in patients with AS[18,27,28]. LVH is also associated with a lower ischemic threshold as a result of capillary rarefaction[29] and transmural steal (with disproportionately high subepicardial blood flow). A higher cut-off value level of FFR to indicate myocardial ischemia is therefore appropriate in patients with AS[30,31].

Although data are scarce, two recent publications on the role of fractional flow reserve (FFR) and instantaneous wave-free ratio (iFR) in the setting of AS and epicardial coronary disease provide important insights. One study found that diagnostic accuracy of iFR was significantly lower in patients with AS when the standard iFR threshold of 0.89 (to correlate with FFR 0.8) was used[28]. The authors found that the best iFR threshold to predict an FFR ≤0.8 in the setting of AS was 0.83 (although iFR values were widely scattered). Another study found that iFR was not subject to change after TAVI (p=0.94) unlike FFR which fell significantly after intervention (p=0.0008)[32]. Positive FFR values worsen after TAVI whilst negative FFR values tend to improve[33].

References:

1. Fallen EL, Elliott WC, Gorlin R. Mechanisms of angina in aortic stenosis. *Circulation*. 1967;36:480–488.

2. Marcus ML, Doty DB, Hiratzka LF, Wright CB, Eastham CL. Decreased coronary reserve: a mechanism for angina pectoris in patients with aortic stenosis and normal coronary arteries. *N Engl J Med*. 1982;307:1362–1366.

3. Omran H, Fehske W, Rabahieh R, Hagendorff A, Lüderitz B. Relation between symptoms and profiles of coronary artery blood flow velocities in patients with aortic valve stenosis: a study using transoesophageal Doppler echocardiography. *Heart*. 1996;75:377–383.

4. Julius BK, Spillmann M, Vassalli G, Villari B, Eberli FR, Hess OM. Angina Pectoris in Patients With Aortic Stenosis and Normal Coronary Arteries: Mechanisms and Pathophysiological Concepts. *Circulation*. 1997;95:892–898.

5. Hildick-Smith DJR, Shapiro LM. Coronary flow reserve improves after aortic valve replacement for aortic stenosis: an adenosine transthoracic echocardiography study. *J Am Coll Cardiol*. 2000;36:1889–1896.

6. Rajappan K, Rimoldi OE, Dutka DP, Ariff B, Pennell DJ, Sheridan DJ, Camici PG. Mechanisms of coronary microcirculatory dysfunction in patients with aortic stenosis and angiographically normal coronary arteries. *Circulation*. 2002;105:470–476.

7. Davies JE, Whinnett ZI, Francis DP, Manisty CH, Aguado-Sierra J, Willson K, Foale RA, Malik IS, Hughes AD, Parker KH, et al. Evidence of a dominant backward-propagating “suction” wave responsible for diastolic coronary filling in humans, attenuated in left ventricular hypertrophy. *Circulation*. 2006;113:1768–1778.

8. Galiuto L, Lotrionte M, Crea F, Anselmi A, Biondi-Zoccai GGL, De Giorgio F, Baldi A, Baldi F, Possati G, Gaudino M, et al. Impaired coronary and myocardial flow in severe aortic stenosis is associated with increased apoptosis: a transthoracic Doppler and myocardial contrast echocardiography study. *Heart.* 2006;92:208–212.

9. Davies JE, Sen S, Broyd C, Hadjiloizou N, Baksi J, Francis DP, Foale RA, Parker KH, Hughes AD, Chukwuemeka A, et al. Arterial pulse wave dynamics after percutaneous aortic valve replacement: fall in coronary diastolic suction with increasing heart rate as a basis for angina symptoms in aortic stenosis. *Circulation*. 2011;124:1565–1572.

10. Steadman CD, Jerosch-Herold M, Grundy B, Rafelt S, Ng LL, Squire IB, Samani NJ, McCann GP. Determinants and functional significance of myocardial perfusion reserve in severe aortic stenosis. *JACC Cardiovasc Imaging*. 2012;5:182–189.

11. Mahmod M, Francis JM, Pal N, Lewis A, Dass S, De Silva R, Petrou M, Sayeed R, Westaby S, Robson MD, et al. Myocardial perfusion and oxygenation are impaired during stress in severe aortic stenosis and correlate with impaired energetics and subclinical left ventricular dysfunction. *J Cardiovasc Magn Reson.* 2014;16:29.

12. Ben-Dor I, Malik R, Minha S, Goldstein SA, Wang Z, Magalhaes MA, Weissman G, Okubagzi PG, Torguson R, Lindsay J, et al. Coronary blood flow in patients with severe

aortic stenosis before and after transcatheter aortic valve implantation. *Am J Cardiol*. 2014;114:1264–1268.

13. Wiegerinck EMA, van de Hoef TP, Rolandi MC, Yong Z, van Kesteren F, Koch KT, Vis MM, de Mol BAJM, Piek JJ, Baan J. Impact of Aortic Valve Stenosis on Coronary Hemodynamics and the Instantaneous Effect of Transcatheter Aortic Valve Implantation. *Circ Cardiovasc Interv*. 2015;8:e002443.

14. Gutierrez-Barrios A, Ruiz-Fernandez D, Gamaza-Chulián S, Agarrado A, Oneto J, Benezet J. TCT-288 Invasive Coronary Microcirculation Assessment In Aortic Stenosis: Index Of Microvascular Resistance. *J Am Coll Cardiol*. 2015;66:B113–B114.

15. Ahn J-H, Kim SM, Park S-J, Jeong DS, Woo M-A, Jung S-H, Lee S-C, Park SW, Choe YH, Park PW, et al. Coronary Microvascular Dysfunction as a Mechanism of Angina in Severe AS: Prospective Adenosine-Stress CMR Study. *J Am Coll Cardiol*. 2016;67:1412–1422.

16. Rolandi MC, Wiegerinck EMA, Casadonte L, Yong Z-Y, Koch KT, Vis M, Piek JJ, Baan J, Spaan JAE, Siebes M. Transcatheter Replacement of Stenotic Aortic Valve Normalizes Cardiac-Coronary Interaction by Restoration of Systolic Coronary Flow Dynamics as Assessed by Wave Intensity Analysis. *Circ Cardiovasc Interv*. 2016;9:e002356.

17. Singh A, Greenwood JP, Berry C, Dawson DK, Hogrefe K, Kelly DJ, Dhakshinamurthy V, Lang CC, Khoo JP, Sprigings D, et al. Comparison of exercise testing and CMR measured myocardial perfusion reserve for predicting outcome in asymptomatic aortic stenosis: the PRognostic Importance of MIcrovascular Dysfunction in Aortic Stenosis (PRIMID AS) Study. *Eur Heart J*. 2017;38:1222–1229.

18. Lumley M, Williams R, Asrress KN, Arri S, Briceno N, Ellis H, Rajani R, Siebes M, Piek JJ, Clapp B, et al. Coronary Physiology During Exercise and Vasodilation in the Healthy Heart and in Severe Aortic Stenosis. *J Am Coll Cardiol*. 2016;68:688–697.

19. Gutierrez-Barrios A, Gamaza-Chulián S, Agarrado-Luna A, Ruiz-Fernandez D, Calle-Pérez G, Marante-Fuertes E, Zayas-Rueda R, Alba-Sánchez M, Oneto-Otero J, Vázquez-Garcia R. Invasive assessment of coronary flow reserve impairment in severe aortic stenosis and echocardiographic correlations. *Int J Cardiol*. 2017;236:370–374.

20. Xaplanteris P, Fournier S, Pijls NHJ, Fearon WF, Barbato E, Tonino PAL, Engstrøm T, Kääb S, Dambrink J-H, Rioufol G, et al, FAME 2 Investigators. Five-Year Outcomes with PCI Guided by Fractional Flow Reserve. *N Engl J Med*. 2018;379:250–259.

21. Fearon WF, Nishi T, De Bruyne B, Boothroyd DB, Barbato E, Tonino P, Jüni P, Pijls NHJ, Hlatky MA, FAME 2 Trial Investigators. Clinical Outcomes and Cost-Effectiveness of Fractional Flow Reserve-Guided Percutaneous Coronary Intervention in Patients With Stable Coronary Artery Disease: Three-Year Follow-Up of the FAME 2 Trial (Fractional Flow Reserve Versus Angiography for Multivessel Evaluation). *Circulation*. 2018;137:480–487.

22. Blumenthal MR, Wang HH, Wang SC. Effect of acute experimental aortic stenosis on coronary circulation. *Circ Res*. 1962;11:727–735.

23. Komaru T, Kanatsuka H, Shirato K. Coronary microcirculation. *Pharmacol Ther*. 2000;86:217–261.

24. Kikoïne J, Lebon M, Gouffran G, Millischer D, Cattan S, Nallet O. [Measurement of fractional flow reserve in patients with severe aortic stenosis: A valid test?]. *Ann Cardiol Angeiol.* 2016;65:366-369.

25. Danson E, Hansen P, Sen S, Davies J, Meredith I, Bhindi R. Assessment, treatment, and prognostic implications of CAD in patients undergoing TAVI. *Nat Rev Cardiol*. 2016;13:276–285.

26. Heusch G. Alpha-adrenergic coronary vasoconstriction in humans. *J Am Coll Cardiol*. 2010;55:1278.

27. Stanojevic D, Gunasekaran P, Tadros P, Wiley M, Earnest M, Mehta A, Lippmann M, Levine M, Dawn B, Gupta K. Intravenous Adenosine Infusion is Safe and Well Tolerated During Coronary Fractional Flow Reserve Assessment in Elderly Patients With Severe Aortic Stenosis. *J Invasive Cardiol*. 2016;28:357-361.

28. Scarsini R, Pesarini G, Zivelonghi C, Piccoli A, Ferrero V, Lunardi M, Barbierato M, Caprioglio F, Vassanelli C, Ribichini F. Coronary physiology in patients with severe aortic stenosis: Comparison between fractional flow reserve and instantaneous wave-free ratio. *Int J Cardiol*. 2017;243:40–46.

29. Krams R, Kofflard MJ, Duncker DJ, Birgelen Von C, Carlier S, Kliffen M, Cate ten FJ, Serruys PW. Decreased coronary flow reserve in hypertrophic cardiomyopathy is related to remodeling of the coronary microcirculation. *Circulation*. 1998;97:230–233.

30. Blows LJ, Redwood SR. The pressure wire in practice. *Heart*. 2007;93:419–422.

31. Di Gioia G, Pellicano M, Toth GG, Casselman F, Adjedj J, Van Praet F, Ferrara A, Stockman B, Degrieck I, Bartunek J, et al. Fractional Flow Reserve-Guided Revascularization in Patients With Aortic Stenosis. *Am J Cardiol*. 2016;117:1511–1515.

32. Ahmad Y, Götberg M, Cook C, Howard J, Malik I, Mikhail G, Frame A, Petraco R, Rajkumar C, Demir O, et al. Coronary Hemodynamics in Patients With Severe Aortic Stenosis and Coronary Artery Disease Undergoing Transcatheter Aortic Valve Replacement: Implications for Clinical Indices of Coronary Stenosis Severity. *JACC Cardiovasc Interv*. 2018;11:2019-2031.

33. Pesarini G, Scarsini R, Zivelonghi C, Piccoli A, Gambaro A, Gottin L, Rossi A, Ferrero V, Vassanelli C, Ribichini F. Functional Assessment of Coronary Artery Disease in Patients Undergoing Transcatheter Aortic Valve Implantation: Influence of Pressure Overload on the Evaluation of Lesions Severity. *Circ Cardiovasc Interv*. 2016;9:1-10.
