# Supplementary material for: Coronary Microcirculation in Aortic Stenosis: A Physiological Hornets’ Nest
Source: Circ Cardiovasc Interv. 2019 Aug 16;12(8):e007547. doi: 10.1161/CIRCINTERVENTIONS.118.007547 (PMC6733603; doi:10.1161/CIRCINTERVENTIONS.118.007547)
Supplement: Supplementary file 2 [file hcv-12-e007547-s002.pdf]

THE AMERICAN PHYSIOLOGICAL SOCIETY LICENSE TERMS AND CONDITIONS

Jul 22, 2019

This Agreement between Hannah ZR McConkey ("You") and The American Physiological Society ("The American Physiological Society") consists of your license details and the terms and conditions provided by The American Physiological Society and Copyright Clearance Center.

All payments must be made in full to CCC. For payment instructions, please see information listed at the bottom of this form.

License Number	4516570144459
License date	Jan 26, 2019
Licensed Content Publisher	The American Physiological Society
Licensed Content Publication	Physiological Reviews
Licensed Content Title	Cross-Talk Between Cardiac Muscle and Coronary Vasculature
Licensed Content Author	Nico Westerhof, Christa Boer, Regis R. Lamberts, et al
Licensed Content Date	Oct 1, 2006
Licensed Content Volume	86
Licensed Content Issue	4
Type of Use	Journal/Magazine
Requestor type	author
Readers being charged a fee for this work	No
Format	print and electronic
Portion	figures/tables/images
Number of figures/tables/images	1
Will you be translating?	no
World Rights	no
Order reference number	
Title of new article	The Coronary Microcirculation in Aortic Stenosis - a physiological hornet's nest
Lead author	HZR McConkey
Title of targeted journal	Circulation: Cardiovascular Interventions
Publisher	Lippincott Williams & Wilkins (LWW)
Expected publication date	Mar 2019
Portions	Figure 18
Requestor Location	Hannah ZR McConkey<br>Cardiovascular Department<br>St Thomas' Hospital<br>Westminster Bridge Road<br>London, London SE1 7EH<br>United Kingdom<br>Attn: Hannah ZR McConkey

Billing Type	Invoice
Billing Address	Hannah ZR McConkey<br>King's College London<br>British Heart Foundation Centre of Excellence<br>Rayne Institute, St. Thomas' Hospital Campus<br>London, United Kingdom SE1 7EH<br>Attn: Hannah ZR McConkey
Total	37.89 GBP

Terms and Conditions

Terms and Conditions:
©The American Physiological Society (APS). All rights reserved. The publisher for this requested copyrighted material is APS. By clicking "accept" in connection with completing this license transaction, you agree to the following terms and conditions that apply to this transaction. At the time you opened your Rightslink account you had agreed to the billing and payment terms and conditions established by Copyright Clearance Center (CCC) available at http://myaccount.copyright.com

The APS hereby grants to you a nonexclusive limited license to reuse published material as requested by you, provided you have disclosed complete and accurate details of your proposed reuse of articles, figures, tables, images, and /or data in new or derivative works. Licenses are for a one-time English language use with a maximum distribution equal to the number of copies identified by you in the licensing process, unless additional options for translations or World Rights were included in your request. Any form of print or electronic republication must be completed within three years from the date hereof. Copies prepared before then may be distributed thereafter

The following conditions are required for a License of Reuse:

Attribution: You must publish in your new or derivative work a citation to the original source of the material(s) being licensed herein, including publication name, author(s), volume, year, and page number prominently displayed in the article or within the figure/image legend.

Abstracts: APS Journal article abstracts may be reproduced or translated for noncommercial purposes without requesting permission, provided the citation to the original source of the materials is included as noted above ("Attribution"). Abstracts or portions of abstracts may not be used in advertisements or commercial promotions.

Non-profit/noncommercial reuse: APS grants permission for the free reuse of APS published material in new works published for educational purposes, provided there is no charge or fee for the new work and/or the work is not directly or indirectly commercially supported or sponsored. Neither original authors nor non-authors may reuse published material in new works that are commercially supported or sponsored including reuse in a work produced by a commercial publisher without seeking permission.

Video and photographs: Some material published in APS publications may belong to other copyright holders and cannot be republished without their permission. The copyright holder of photographs must be ascertained from the original source by the permission requestor. Videos and podcasts may not be rebroadcast without proper attribution and permission as requested here. For further inquiries on reuse of these types of materials, please contact cvillemez@the-aps.org

Figures/Tables/Images are available to the requestor from the APS journals website at http://www.the-aps.org/publications/journals/. The obtaining of content is a separate transaction and does not involve Rightslink or CCC, and is the responsibility of the permission seeker. Higher resolution images are available at additional charge from APS; please contact cvillemez@the-aps.org

Original Authors of Published Works: To see a full list of original authors rights concerning their own published work http://www.the-aps.org/publications/authorinfo/copyright.htm

Content reuse rights awarded by the APS may be exercised immediately upon issuance of

this license, provided full disclosure and complete and accurate details of the proposed reuse have been made; no license is finally granted unless and until full payment is received either by the publisher or by CCC as provided in CCC's Billing and Payment Terms and Conditions. If full payment is not received on a timely basis, then any license preliminarily granted shall be deemed automatically revoked and shall be void as if never granted. Further, in the event that you breach any of these Terms and Conditions or any of CCC's Billing and Payment Terms and Conditions, the license is automatically revoked and shall be void as if never granted. Use of materials as described in a revoked license, as well as any use of the materials beyond the scope of the license, may constitute copyright infringement and the Publisher reserves the right to take action to protect its copyright of its materials.
The APS makes no representations or warranties with respect to the licensed material. You hereby indemnify and agree to hold harmless the publisher and CCC, and their respective officers, directors, employees and agents, from and against any and all claims arising out of your use of the licensed material other than as specifically authorized pursuant to this license.
This license is personal to you /your organization and may not be sublicensed, assigned, or transferred by you /your organization to another person /organization without the publisher's permission.This license may not be amended except in writing signed by both parties, or in the case of the publisher, by CCC on the publisher's behalf.
The APS reserves all rights not specifically granted in the combination of (i) the license details provided by you and accepted in the course of this licensing transaction, (ii) these Terms and Conditions and (iii) CCC's Billing and Payment Terms and Conditions.
v1.0

You will be invoiced within 48 hours of this transaction date. You may pay your invoice by credit card upon receipt of the invoice for this transaction. Please follow instructions provided at that time.
To pay for this transaction now; please remit a copy of this document along with your payment. Payment should be in the form of a check or money order referencing your account number and this invoice number RLNK502986609.

Make payments to "COPYRIGHT CLEARANCE CENTER" and send to:

Copyright Clearance Center
29118 Network Place
Chicago, IL 60673-1291
Please disregard electronic and mailed copies if you remit payment in advance

Questions? customercare@copyright.com or +1-855-239-3415 (toll free in the US) or +1-978-646-2777.
