# Supplementary material for: Coronary Microcirculation in Aortic Stenosis: A Physiological Hornets’ Nest
Source: Circ Cardiovasc Interv. 2019 Aug 16;12(8):e007547. doi: 10.1161/CIRCINTERVENTIONS.118.007547 (PMC6733603; doi:10.1161/CIRCINTERVENTIONS.118.007547)
Supplement: Supplementary file 6 [file hcv-12-e007547-s006.pdf]

OXFORD UNIVERSITY PRESS LICENSE TERMS AND CONDITIONS

Jul 22, 2019

This Agreement between Hannah ZR McConkey ("You") and Oxford University Press ("Oxford University Press") consists of your license details and the terms and conditions provided by Oxford University Press and Copyright Clearance Center.

All payments must be made in full to CCC. For payment instructions, please see information listed at the bottom of this form.

License Number	4597231139341
License date	May 27, 2019
Licensed Content Publisher	Oxford University Press
Licensed Content Publication	European Heart Journal
Licensed Content Title	Coronary microvascular dysfunction: an update
Licensed Content Author	Crea, Filippo; Camici, Paolo G.
Licensed Content Date	Dec 23, 2013
Licensed Content Volume	35
Licensed Content Issue	17
Type of Use	Journal
Requestor type	Educational Institution/Non-commercial/ Not for-profit
Pharmaceutical support or sponsorship for this project	No
Format	Electronic
Portion	Figure/table
Number of figures/tables	1
Will you be translating?	No
Circulation/distribution	5000
Title of new article	The Coronary Microcirculation in Aortic Stenosis - a physiological hornet's nest
Lead author	HZR McConkey
Title of targeted journal	Circulation: Cardiovascular Interventions
Publisher	Lippincott Williams & Wilkins (LWW)
Expected publication date	Jun 2019
Portions	Table 1
Requestor Location	Eleni Vomvyla<br>Library Services<br>Maughan Library<br>Chancery Lane<br>London, London WC2A 1LR<br>United Kingdom<br>Attn: Hannah ZR McConkey
Publisher Tax ID	GB125506730
Billing Type	Invoice

Billing Address	Hannah ZR McConkey<br>King's College London<br>British Heart Foundation Centre of Excellence<br>Rayne Institute, St. Thomas' Hospital Campus<br>London, United Kingdom SE1 7EH<br>Attn: Hannah ZR McConkey
Price	55.77 GBP
Tax/VAT (20%)	11.15 GBP
Total	66.92 GBP

Terms and Conditions

STANDARD TERMS AND CONDITIONS FOR REPRODUCTION OF MATERIAL FROM AN OXFORD UNIVERSITY PRESS JOURNAL

1. Use of the material is restricted to the type of use specified in your order details.
2. This permission covers the use of the material in the English language in the following territory: world. If you have requested additional permission to translate this material, the terms and conditions of this reuse will be set out in clause 12.
3. This permission is limited to the particular use authorized in (1) above and does not allow you to sanction its use elsewhere in any other format other than specified above, nor does it apply to quotations, images, artistic works etc that have been reproduced from other sources which may be part of the material to be used.
4. No alteration, omission or addition is made to the material without our written consent. Permission must be re-cleared with Oxford University Press if/when you decide to reprint.
5. The following credit line appears wherever the material is used: author, title, journal, year, volume, issue number, pagination, by permission of Oxford University Press or the sponsoring society if the journal is a society journal. Where a journal is being published on behalf of a learned society, the details of that society must be included in the credit line.
6. For the reproduction of a full article from an Oxford University Press journal for whatever purpose, the corresponding author of the material concerned should be informed of the proposed use. Contact details for the corresponding authors of all Oxford University Press journal contact can be found alongside either the abstract or full text of the article concerned, accessible from www.oxfordjournals.org Should there be a problem clearing these rights, please contact journals.permissions@oup.com
7. If the credit line or acknowledgement in our publication indicates that any of the figures, images or photos was reproduced, drawn or modified from an earlier source it will be necessary for you to clear this permission with the original publisher as well. If this permission has not been obtained, please note that this material cannot be included in your publication/photocopies.
8. While you may exercise the rights licensed immediately upon issuance of the license at the end of the licensing process for the transaction, provided that you have disclosed complete and accurate details of your proposed use, no license is finally effective unless and until full payment is received from you (either by Oxford University Press or by Copyright Clearance Center (CCC)) as provided in CCC's Billing and Payment terms and conditions. If full payment is not received on a timely basis, then any license preliminarily granted shall be deemed automatically revoked and shall be void as if never granted. Further, in the event that you breach any of these terms and conditions or any of CCC's Billing and Payment terms and conditions, the license is automatically revoked and shall be void as if never granted. Use of materials as described in a revoked license, as well as any use of the materials beyond the scope of an unrevoked license, may constitute copyright infringement and Oxford University Press reserves the right to take any and all action to protect its copyright in the materials.
9. This license is personal to you and may not be sublicensed, assigned or transferred by you to any other person without Oxford University Press's written permission.

10. Oxford University Press reserves all rights not specifically granted in the combination of (i) the license details provided by you and accepted in the course of this licensing transaction, (ii) these terms and conditions and (iii) CCC's Billing and Payment terms and conditions.
11. You hereby indemnify and agree to hold harmless Oxford University Press and CCC, and their respective officers, directors, employs and agents, from and against any and all claims arising out of your use of the licensed material other than as specifically authorized pursuant to this license.
12. Other Terms and Conditions:
v1.4

You will be invoiced within 48 hours of this transaction date. You may pay your invoice by credit card upon receipt of the invoice for this transaction. Please follow instructions provided at that time.
To pay for this transaction now; please remit a copy of this document along with your payment. Payment should be in the form of a check or money order referencing your account number and this invoice number RLNK503132578.

Make payments to "COPYRIGHT CLEARANCE CENTER" and send to:

Copyright Clearance Center
29118 Network Place
Chicago, IL 60673-1291
Please disregard electronic and mailed copies if you remit payment in advance

Questions? customercare@copyright.com or +1-855-239-3415 (toll free in the US) or +1-978-646-2777.
